# Monitoring of CO_2_ Absorption Solvent in Natural Gas Process Using Fourier Transform Near-Infrared Spectrometry

**DOI:** 10.1155/2020/9830685

**Published:** 2020-02-07

**Authors:** Mohd Yusop Nurida, Dolmat Norfadilah, Mohd Rozaiddin Siti Aishah, Chan Zhe Phak, Syafiqa M. Saleh

**Affiliations:** ^1^PETRONAS Research Sdn. Bhd., Lot 3288 & 3289, Off Jalan Ayer Itam, Kawasan Institusi Bangi, Kajang 43000, Selangor, Malaysia; ^2^Universiti Teknologi Petronas, Seri Iskandar 32610, Perak, Malaysia

## Abstract

The analytical methods for the determination of the amine solvent properties do not provide input data for real-time process control and optimization and are labor-intensive, time-consuming, and impractical for studies of dynamic changes in a process. In this study, the potential of nondestructive determination of amine concentration, CO_2_ loading, and water content in CO_2_ absorption solvent in the gas processing unit was investigated through Fourier transform near-infrared (FT-NIR) spectroscopy that has the ability to readily carry out multicomponent analysis in association with multivariate analysis methods. The FT-NIR spectra for the solvent were captured and interpreted by using suitable spectra wavenumber regions through multivariate statistical techniques such as partial least square (PLS). The calibration model developed for amine determination had the highest coefficient of determination (*R*^2^) of 0.9955 and RMSECV of 0.75%. CO_2_ calibration model achieved *R*^2^ of 0.9902 with RMSECV of 0.25% whereas the water calibration model had *R*^2^ of 0.9915 with RMSECV of 1.02%. The statistical evaluation of the validation samples also confirmed that the difference between the actual value and the predicted value from the calibration model was not significantly different and acceptable. Therefore, the amine, CO_2_, and water models have given a satisfactory result for the concentration determination using the FT-NIR technique. The results of this study indicated that FT-NIR spectroscopy with chemometrics and multivariate technique can be used for the CO_2_ solvent monitoring to replace the time-consuming and labor-intensive conventional methods.

## 1. Introduction

Natural gas is referring to the fossil fuel gas that is found in the oil and/or gas fields [1]. Natural gas consists primarily of methane (CH_4_) with a trace amount of contaminants such as CO_2_, H_2_S, water vapor, N_2_, and He. CO_2_ and H_2_S are considered the most significant and impactful contaminants as CO_2_ is a greenhouse gas that lowers efficiency in production, transportation, and storage and causes major climate change, while H_2_S has a pungent odor, is poisonous, and causes corrosion to equipment and pipelines [2].

Natural gas needs to be treated to remove the contaminants to meet the pipeline transport specification. The CO_2_ content in sales natural gas has to be lower than 2 vol.%. This makes the removal of CO_2_ from natural gas necessary [3, 4]. For a conventional gas processing, CO_2_ is removed in an acid gas removal unit (AGRU) using aqueous solvent absorption processes [5]. Absorption processes with chemical solvents are currently the most used technology for CO_2_ separation from natural gas commercially as they are much more efficient and cost-effective compared with other processes [6]. Several solvents have been proposed for CO_2_ absorption, but the common solvent used are the alkanolamines such as monoethanolamine (MEA), diethanolamine (DEA), diisopropanolamine (DIPA), and methyldiethanolamine (MDEA) [7].

There are two important parameters for the solvent-based CO_2_ removal process: the concentrations of the active solvent component and the dissolved acid gas. For commercial application, the CO_2_ absorption solvent does not only contain active solvent, absorbed gas, and water, but also contain heat stable salts and solvent degradation products [8]. The data on the CO_2_ loading (mol CO_2_/mol amine) and active ingredient concentration of solvents and water content are essential for the CO_2_ solvent absorption operation monitoring and in various experimental conditions studying CO_2_ absorption. Conventionally, this solvent analysis was performed manually by the use of multiple analytical methods for different parameters which are relatively labor-intensive and time-consuming [9]. Currently, the most popular analytical method for determining the acid gas concentration in amine solvent uses wet chemistry titration. The water content is most often determined by the Karl Fischer method. The wet chemistry titration has a number of serious disadvantages. The total analysis time required could be as long as two hours. During this time, the CO_2_/amine solution is open to the atmospheric pressure so both flashing of CO_2_ and degradation of the amine can occur [10]. Manual off-line liquid sampling and analysis does not provide input data for real-time process control and optimization. Furthermore, the methods used are mostly impractical for studies of dynamic changes in a process [9].

Process analytical technology (PAT) is a system for analysis, monitoring, and control of the critical parameters of the operational process. PAT includes scientifically based process design that identifies key and critical operational parameters, appropriate measurement devices, statistical information technology tools, and feedback process control strategies that work together to ensure the effectiveness of the process. For several years, Fourier transform near-infrared (FT-NIR) spectroscopy has become a PAT of great interest because it is a rapid, nondestructive technique and does not utilize toxic solvent or reagents which make it one of the most favorable spectroscopic techniques [11]. The advantage of choosing FT-NIR as a quantitative technique lies in its ability to readily carry out multicomponent analysis in association with multivariate analysis methods such as partial least squares (PLS) regression [9].

Chemometrics is the study of both mathematical and statistical methods that are used to maximize the information and data obtained from experiments. A regression analysis is when a statistic model is established to correlate the relationship between a measured parameter and a variation of other independent variables [12]. When a regression model is created for the calibration model, the spectral points in the measured spectra are used to compute the unknown variables. For further enhancement, optimization of the spectral range can be done by describing the concentration of the sample and excluding the regions that are created by noise [13].

Overall, the process of developing a calibration model would require a significant amount of samples so that sufficient spectrum can be used to create a mathematical model. The sample measurement sets consist of the calibration set and validation set. The general steps taken in preparing this model are as follows: (1) data collection: preparing samples with targeted composition by collecting and analyzing the sample sets using a reference method; (2) spectra collection: measuring the spectra in the FT-NIR spectroscopy; (3) establishing a calibration model along with optimizing the model using the FT-NIR spectroscopy; and (4) validating the model using validation sample sets to verify the accuracy and repeatability of the model.

The objective of this work is to develop a method for monitoring the CO_2_ solvent in the natural gas processing using FT-NIR technique. A robust calibration model will be developed for the solvent analysis with different solvent concentrations, CO_2_ loading, and water content.

## 2. Experimental

Multivariate quantitative analysis using FT-NIR consists of some steps including selection of sample for calibration set, reference method analysis or determination of the properties of samples using reference method, collection of spectroscopic data, development of the prediction model using reference method's data and spectra collected, and validation of the developed calibration model.

### 2.1. CO_2_ Solvent Absorption Samples

MDEA (≥98%) was purchased from Sigma-Aldrich and used as received without further purification. Aqueous solutions of amines were prepared by mixing the amine with deionized water. Compressed CO_2_ from a gas cylinder (≥99%) was used to load the stock solutions. Different sets of calibration samples were prepared comprising of amine, CO_2_, and water. The concentrations of amine in the samples vary from 20 wt.% to 60 wt.%, and each amine concentration comprises a CO_2_ content between 1 and 10 wt.%, respectively.

### 2.2. Solvent Analysis

#### 2.2.1. Amine Concentration

The amine in the prepared samples was analyzed by the titration technique based on the ASTM D3590-17 method. The amine concentration was determined by titrating the sample against 0.50 N HCl. 4.5 g sample of the prepared solution was weighed using a weight balance with record up to ±0.0001 g. The solution was then diluted with water to 100 mL and then titrated against 0.5 N HCl until pH 4.5. The sample weight and amount of HCl used were recorded:

(1) $$\begin{array}{c} \text{Amine content}=\frac{\left(\text{volume of HCl used}\times \text{HCl normality}\times 9.102\right)}{\left(\text{sample weight}\right)}. \end{array}$$

#### 2.2.2. CO_2_ Determination

The CO_2_ content in the prepared samples was analyzed by the titration technique based on the UOP Method 829. 20 g of sample solution was weighed into a beaker using a weight balance with record up to ±0.0001 g. 120 mL of methanol that has been pH-adjusted to 11.20 was added into the sample solution. The mixture was titrated against 0.5 N KOH until pH 11.20 was attained.

(2) $$\begin{array}{c} {\text{CO}}_{2}\text{ content}=\left[\frac{\left(\text{volume of KOH used}\right)}{\left(\left(\text{sample weight}\right)-0.0249\right)}\right]\times \text{KOH normality}\times 4.4. \end{array}$$

#### 2.2.3. Water Content

The water content in the samples was determined using Mettler Toledo Karl Fischer instrument model V10S.

### 2.3. Collection of Spectroscopic Data

FT-NIR spectra for the same sample that have been used in the reference method were analyzed using ABB MB3000 spectrometers with Harrick cell setup with the path length of 0.5 mm. The spectra were captured at resolution 32 cm^−1^ with 16 scans at the range of 700 to 4000 cm^−1^.

### 2.4. Preprocessing of Spectral Data

**Figure 1:**
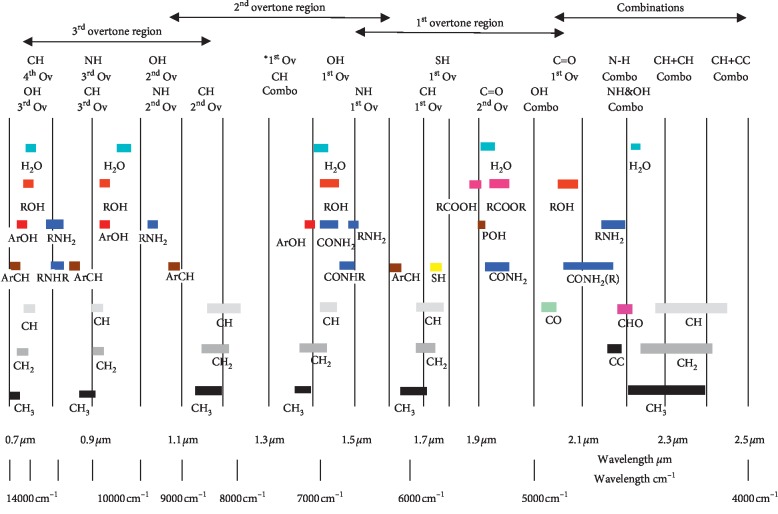
Absorption band in near-infrared (NIR) [14].

The selection of spectra region depends on the range of specific functional group as per Figure 1 and also the correlation between spectra and reference method results. Preprocessing of spectra is a step where spectra have been processed or treated to increase the influence of significant spectral regions in contrast to nonsignificant parts. There are a lot of pretreatment methods that can be used which include baseline correction, thickness correction, standard normal variate (SNV), and many more. In this study, baseline correction was used to preprocess the collected spectra. Baseline correction is used to eliminate the baseline offset. The spectra data were mean-centred to get an average spectrum before one of the preprocessing methods above was applied. The model was validated using full cross validation where it considers all samples during the calculation of the calibration model.

### 2.5. Multivariate Analysis

The quantitative prediction model was developed using the partial least square (PLS) method in HORIZON software. In the PLS method, collected spectra from the calibration set analysis were correlated with the reference method's data to predict the value of unknown samples. The chemometric parameters such as coefficient of determination (*R*^2^) and the root-mean-square error of cross validation (RMSECV) indicates the effectiveness of the developed prediction model.

The coefficient of determination (*R*^2^) is the square of the correlation between the predicted data point and the actual data point. It can be represented as the equation below where *y*_*i*_ represents the data point and how much they vary around the mean, *y*_m_:

(3) $$\begin{array}{c} R^{2}=\left(1-\frac{\text{SSE}}{\sum {\left(y_{i}-y_{\text{m}}\right)}^{2}}\right). \end{array}$$

The root-mean-square error calibration (RMSEC) is developed from an average of how close data points are at the calibration line. The root-mean-square error cross validation (RMSECV) on the other hand is a method to test the calibration model that has been developed. It can help in ensuring the developed model is not overfitted and also in determining the outliers. Here, a data point or spectrum of a sample is removed from the calibration set and the model is developed with the remaining spectra. The process is repeated with removing and inserting back the sample where a new RMSEC is developed. All the RMSEC collected will then be averaged which will give the overall RMSECV. The RMSECV can also be determined by the following equation as mentioned by Liu et al. [15]:

(4) $$\begin{array}{c} \text{RMSECV}=\sqrt{\frac{\sum_{i=1}^{n}{\left(x_{i}-y_{i}\right)}^{2}}{n}}, \end{array}$$

where *n* is the number of samples and *x*_*i*_ is the results that are obtained by the NIR method.

After building the calibration model, the software develops a calibration function and soon the model has to be tested on its reliability of prediction. The spectra collected from the NIR correspond to the concentration of the sample and the data are randomly distributed into both the calibration set and datasets. The calibration data are used to develop the PLS regression calibration model whereas the validation data are used to validate the model performance.

High *R*^2^, low RMSECV, low spectral residual, and a small difference between the RMSEC and RMSECV (Ref) lead to excellent prediction model. Spectra residual is a difference value between the actual and predicted data. Besides, to minimize the risk of error, the outlier was eliminated from the calibration set. Validation samples were prepared and tested to verify the prediction model reliability.

## 3. Results and Discussion

### 3.1. Reference Method Analysis: Amine, CO_2_, and Water Content

The common range of amine solvent concentration used in gas processing plants is between 30 and 60 wt.% [5]. The concentration range of amine solvent chosen for this study was between 20 and 60 wt.% to include the same range of solvent concentration used by the gas processing plants. The analysis results of solvent samples for amine, CO_2_, and water content using the reference method are listed in Table 1.

### 3.2. Spectral Data Collection

**Figure 2:**
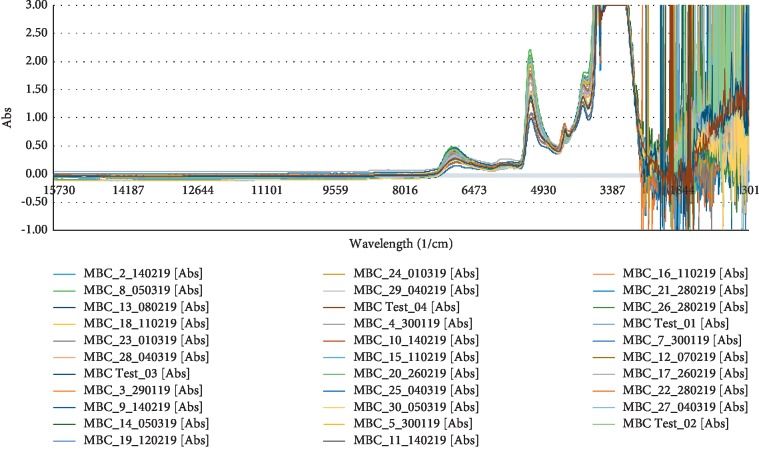
Raw FT-NIR absorbance spectra recorded for solvent samples.

30 samples with 20 wt.% to 60 wt.% concentration of amine and CO_2_ content of 1, 3, 5, 6, 8, and 10 wt.% were selected in the calibration set. The spectra of each sample were collected using ABB FT-NIR and processed using ABB HORIZON software as indicated in Figure 2. The important steps involved after measuring the spectra are selecting the interactive region and spectra preprocessing. As mentioned by Zhang and Su [16], the importance of selecting the wavelength range is to further improve the accuracy of the results. Therefore, it is important to select regions of positive correlations and reject the negative. The next is spectra preprocessing where it has been used to reduce noise and extract useful information for developing a quantitative calibration model where it includes smoothing, normalization, scatter-correction, and derivatives [17]. This spectral pretreatment will help to lower the errors of estimation for the calibration model developed.

**Figure 3:**
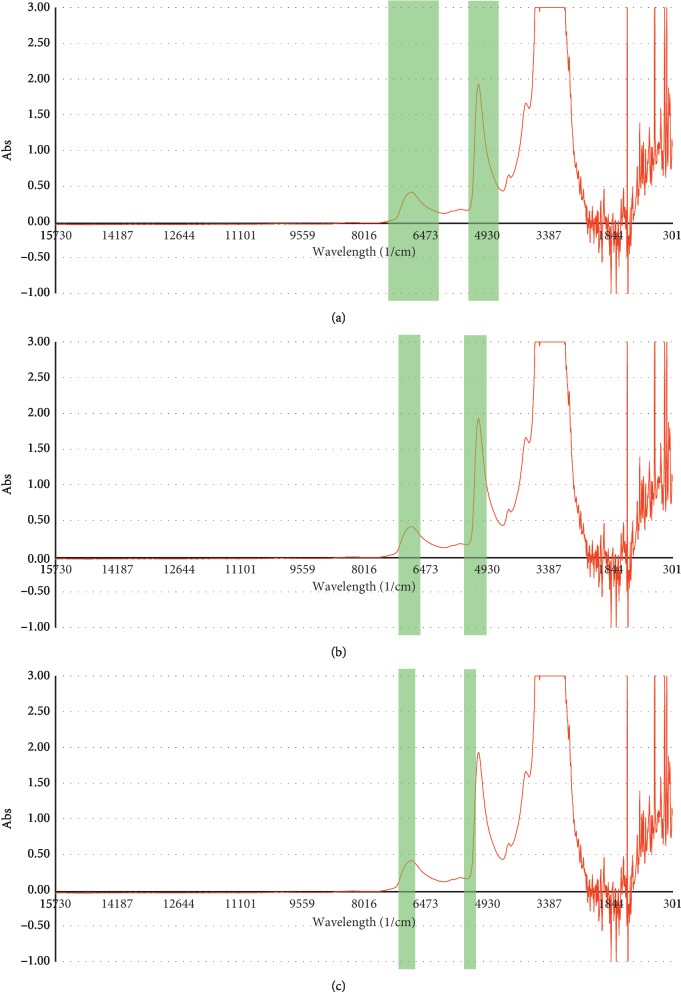
Selected region for (a) amine, (b) CO_2_, and (c) water calibration model.

The region below 3800 cm^−1^ was seen to be saturated and so it is removed from the spectral selection region. The spectrum region for amine, CO_2_, and water was selected according to the functional group and correlation spectrum as per Figure 3. The spectral region selection for amine is 4543.96–5261.42 and 6248.91–7143.81 cm^−1^, whereas for CO_2_, the region selected was 4999.12–5554.58 and 6665.50–7143.81 cm^−1^. For water, the spectra range from 5261.42 to 5400.29 and 6796.65 to 7143.81 cm^−1^. In the spectral range that has been chosen, ketone and alcohols are likely to be the source of interference for the spectrum. Fortunately, the solvent samples used do not contain any of the interfering compounds.

### 3.3. Calibration Model Development

The calibration sample undergone similar preprocessing method of baseline correction since other preprocessing methods showed no improvement in the quality of the prediction model.  Table 2 shows the result of the multivariate analysis for the calibration samples. HORIZON software has automatically proposed the number of factors for which the model reached its minimum performance; however, too high number of factor will interpret spectral noise and thus reduces the quality of the calibration model.

**Figure 4:**
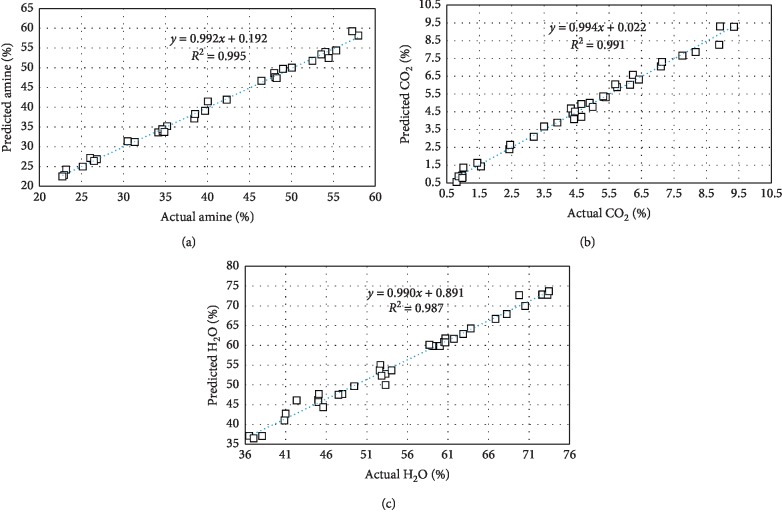
Calibration model and coefficient of determination (*R*^2^) for (a) amine, (b) CO_2_, and (c) water.

Based on the results from Table 2, three different calibration models were established with baseline correction and region selection. The predictive values versus the measured values are depicted in Figure 4. The calibration model for amine determination had the highest coefficient of determination (*R*^2^) of 0.9955 and RMSECV of 0.75%. CO_2_ calibration model achieved *R*^2^ of 0.9902 with RMSECV of 0.25% whereas the water calibration model had *R*^2^ of 0.9915 with RMSECV of 1.02%. Therefore, it can be seen from Figure 4 that amine, CO_2_, and water models have given a satisfactory result for the concentration determination using the FT-NIR technique. Since the testing was conducted with samples that covered a broad range of amine concentration (30–60%), the accuracy of the model is considered sufficient. Based on the above results, the three models were reliable and could accurately predict the solvent quality and can be monitored via FT-NIR technique using the developed quantitative models. As this technique was successful in determining the solvent quality, there is also the potential for the same technique to be developed to determine the natural gas feed concentration.

### 3.4. Validation of the Predicted Calibration Model

**Figure 5:**
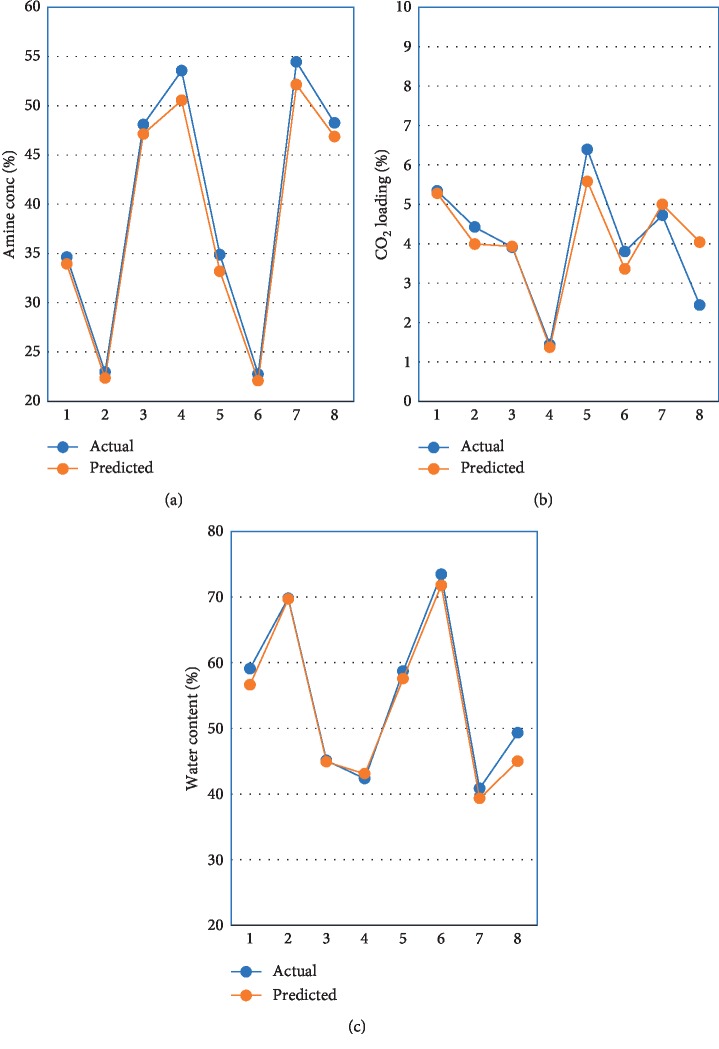
Actual versus predicted values for (a) amine, (b) CO_2_, and (c) water content.

Validation samples were tested in order to verify the accuracy of the developed prediction model. The plot of actual versus predicted values calculated for the calibration model is depicted in Figure 5. Difference between the actual and predicted values for validation sample was evaluated using *t*-test statistical method and the results are listed in Table 3.

Results of *t*-test statistical evaluation showed *t*-calculated for amine, CO_2_, and water content was less than *t* critical. Statistical evaluation confirmed that the difference between the actual value and predicted value from the calibration model was not significantly different and acceptable.

## 4. Conclusion

The present work has demonstrated that FT-NIR spectroscopy can be a suitable technique for CO_2_ solvent monitoring in the gas processing unit. The use of FT-NIR spectral information and multivariate techniques showed potential for the simultaneous detection of multiple components in the CO_2_ solvent system. The developed method can also be used to design the online measurement of the CO_2_ solvent monitoring system to obtain the real-time data of the solvent conditions.

## Figures and Tables

**Table 1:** Amine, CO_2_, and water content in the prepared solvent sample.

Sample	Actual composition, wt.%			mol CO_2_/mol amine
	Amine	CO_2_	Water	
1	57.25	4.65	38.10	0.21
2	55.33	8.17	34.03	0.38
3	54.03	8.92	33.47	0.42
4	50.28	2.58	41.38	0.13
5	49.21	0.69	42.45	0.04
6	48.66	0.69	43.88	0.04
7	47.96	6.42	40.58	0.34
8	47.11	1.55	47.62	0.08
9	46.45	4.73	46.32	0.26
10	45.72	0.97	53.30	0.05
11	40.81	3.80	50.65	0.24
12	40.26	6.96	49.29	0.44
13	40.05	7.13	50.36	0.46
14	35.05	1.07	63.10	0.08
15	34.73	3.26	62.69	0.24
16	33.49	5.10	54.44	0.39
17	32.96	5.81	59.15	0.45
18	32.52	4.99	60.01	0.39
19	31.95	5.69	56.48	0.46
20	31.35	8.49	55.83	0.69
21	30.45	9.36	48.78	0.79
22	29.43	2.90	67.28	0.25
23	27.76	4.59	64.27	0.42
24	27.60	4.45	56.99	0.41
25	26.95	4.65	60.01	0.44
26	23.20	3.50	73.30	0.39
27	21.83	3.183	58.32	0.37
28	21.42	6.15	67.98	0.73
29	20.69	6.72	75.01	0.83
30	20.08	0.64	79.28	0.08

**Table 2:** Range, *R*^2^, RMSECV, and factor for different models of amine, CO_2_, and water.

Model	Range	*R* ^*2*^	RMSECV	Factor
Amine	4543.96–5261.42,	99.55	0.75	4
	6248.91–7143.81 cm^−1^			

CO_2_	4999.12–5554.58,	99.02	0.25	5
	6665.50–7143.81 cm^−1^			

Water	5261.42–5400.29,	99.15	1.02	4
	6796.65–7143.81 cm^−1^			

**Table 3:** Difference between the actual and predicted values for validation sample evaluated using *t*-test (paired two sample for means).

	Amine		CO_2_		Water	
	Actual	Predicted	Actual	Predicted	Actual	Predicted
Mean	39.2875	53.5	4.0575	4.11125	54.83125	54.86125
Variance	158.7985	154.0814	2.458764	1.723298	154.4134	153.2284
Observations	8	8	8	8	8	8
Pearson correlation	−0.98999		0.890078		0.990796	
Hypothesized mean difference	0		0		0	
Df	7		7		7	
***t* Calc**	**−1.61106**		**−0.21129**		**−0.05041**	
*P* (*T* ≤ *t*), one-tailed	0.0756		0.41934		0.480604	
*t* critical, one-tailed	1.894579		1.894579		1.894579	
*P* (*T* ≤ *t*), two-tailed	0.151201		0.838681		0.961207	
***t* critical, two-tailed**	**2.364624**		**2.364624**		**2.364624**	

## Data Availability

The data used to support the findings of this study are available from the corresponding author upon request.
